# Comparison of the pressure-relieving properties of various types of forefoot pads in older people with forefoot pain

**DOI:** 10.1186/1757-1146-6-S1-O19

**Published:** 2013-05-31

**Authors:** Pei Y Lee, Karl B Landorf, Daniel R Bonanno, Hylton B Menz

**Affiliations:** 1 Department of Podiatry, Singapore General Hospital, Singapore; 2 Department of Podiatry, La Trobe University, Melbourne, Australia; 3 Lower Extremity and Gait Studies Program, La Trobe University, Melbourne, Australia

## Background

Plantar forefoot pain is commonly experienced by older people and it is often treated with forefoot pads. However, studies have reported inconsistent results for the effectiveness of different pads on plantar pressure reduction, and optimum pad placement is still not clear. The aim of this study was to compare the effects of different forefoot pads on plantar pressures under the forefoot in older people with forefoot pain.

## Methods

Thirty-seven adults with current or previous forefoot pain and a mean age of 73.5 (SD 4.8) participated. In-shoe forefoot plantar pressure data were recorded using the pedar^®^-X while participants walked along an 8 m walkway wearing a standardised shoe and four different forefoot padding conditions; (i) metatarsal dome positioned 10 mm proximal to the metatarsal heads, (ii) metatarsal dome positioned 5 mm distal to the metatarsal heads, (iii) metatarsal bar, and (iv) plantar cover.

## Results

Compared to the shoe-only condition, each of the forefoot pads significantly reduced forefoot peak pressure and maximum force. The plantar cover and the metatarsal dome positioned 5 mm distal to the metatarsal heads were most effective in reducing peak pressure (19%, p<0.001 and 18%, p<0.001, respectively).

## Conclusion

These findings indicate that forefoot pads are effective in reducing forefoot pressures in older people with forefoot pain, and that the fore/aft position of the pad relative to the metatarsal heads may be more important than the shape of the pad.

